# Zein-CMC-PEG Multiple Nanocolloidal Systems as a Novel Approach for Nutra-Pharmaceutical Applications

**DOI:** 10.15171/apb.2019.030

**Published:** 2019-06-01

**Authors:** Afshin Babazadeh, Mahnaz Tabibiazar, Hamed Hamishehkar, Bingyang Shi

**Affiliations:** ^1^Motor Neuron Disease Research Center, Department of Biomedical Sciences, Faculty of Medicine and Health Sciences, Macquarie University, Sydney, NSW 2109, Australia.; ^2^Nutrition Research Center and Department of Food Science and Technology, Faculty of Nutrition and Food Science and Technology, Tabriz University of Medical Sciences, Tabriz, Iran.; ^3^Drug Applied Research Center, Tabriz University of Medical Sciences, Tabriz, Iran.

**Keywords:** Carboxymethyl cellulose, Nanoencapsulation, Protein-polysaccharide complex, Rutin, Zein nanoparticles

## Abstract

***Purpose:*** Hydrophobic nutraceuticals are suffering from water solubility and physicochemical stabilities once administered to the body or food matrixes. The present study depicts the successful formulation of a zein-carboxymethyl cellulose (CMC) complex to stabilize a water in oil (W/O) emulsion to protect them from environmental and gastrointestinal conditions. The formulated water in oil in water (W/O/W) system was used for nanoencapsulating of hydrophobic nutraceutical, rutin, via protein-polysaccharide complexes.

***Methods:*** Zein nano particles smaller than 100 nm were produced using poly ethylene glycol (PEG 400) and Tween 80, which eliminates the use of ethanolic solutions in preparation of zein nanoparticles (ZN). CMC was then added to the ZN under magnetic stirrer to provide zein-CMC complex. A concentration of 20% CMC showed the smallest particle size (<100 nm). Rutin was dispersed in water in oil in water (W/O/W) emulsion stabilized by zein-CMC complex. A set of experiments such as encapsulation efficiency (EE%), encapsulation stability (ES%), and releasing rate of rutin were measured during 30 days of storage at 4°C.

***Results:*** Results showed that, produced multiple emulsion prepared with lower concentrations of Tween 80 (0.5%), ethanol: PEG: water ratio of 0:80:20 showed smaller size (89.8±4.2 nm). ES% at pH values of 1.2, 6.8, and 7.4 were 86.63±6.19, 91.54±3.89, and 97.13±2.39 respectively, indicating high pH tolerability of formulated W/O/W emulsions.

***Conclusion:*** These findings could pave a new approach in stabilizing W/O/W emulsions for encapsulating and controlling the release of water insoluble nutraceuticals/drugs.

## Introduction

Nutraceuticals play an important role in providing more health and make the body to defeat against the radical agents produced from different living conditions and diet habit. The health promoting effects of herbal compounds such as rutin, resveratrol, and so on were well documented.^1,2^ In spite of various disease curing and health promoting effects of herbal compounds such as rutin, they are unstable compounds and can easily get degraded.^3^ Therefore, encapsulating these valuable nutraceuticals not only can protect them from undesired environmental and gastrointestinal conditions, but also make it possible to design new nano-based medicinal-functional foods.^4,5^ Different types of encapsulating systems can be used for these purposes. The protein-polysaccharide complexes have been applied in developing stable emulsions.^6^ Interactions between proteins and polysaccharides could provide new complexes with new interfacial behaviors, which can be used in food and pharmaceutical sciences. Covalent bonds and electrostatic interactions are the most common interactions occurs between proteins and polysaccharides. These interactions can be influenced by ionic strength, pH, and charge distribution on biopolymers affecting the final protein-polysaccharide complex integrity.^7^ The structural integrity and stabilizing features of a protein-polysaccharide complex can also be affected by the sequence of biopolymers, which were adsorbed onto the interface of two immiscible phases.^8^ In protein-polysaccharide complexes, proteins behave as the main stabilizer component and polysaccharides involved in stabilizing emulsions via their viscosity-enhancing features in the aqueous phase and consequently improving the interfacial membrane stability due to their thickening and steric stabilizing properties.^9^ Carboxymethyl cellulose (CMC) was introduced as an imperative industrial polymer with various applications in food and pharmaceutical sciences.^10,11^ The most important feature of CMC is its viscosity building function. CMC is easily available and also is a very cheap among all the polysaccharides, which makes it a good candidate for industrial scale production of food formulations. Various researches have shown the advantages of protein-polysaccharides complexes to stabilize emulsions by their capability to engineer the interfacial membranes.^12^

During last decade, various types of emulsions such as lipid based delivery systems (i.e nanostructured lipid carriers, liposomes, and etc) have been studied in the delivery of nutraceuticals in food and pharmaceutical sciences.^13-16^ Proteins (e.g., zein) are natural emulsifiers and stabilizers that can produce thermodynamically and kinetically stable emulsions by providing a viscoelastic layer once they absorbed onto the oil droplets.^17^ Pickering emulsions are defined as emulsions stabilized by solid particles, which could be used as a texture modification, reduction in calorie and fat content, and nutraceutical encapsulation.^18^ In comparison to emulsions stabilized by surfactants or biopolymers, pickering emulsions are known to show a long-term stability against instability phenomena such as coalescence and Ostwald ripening. Pickering emulsions are able to stabilize o/w emulsions via their have amphiphilic behaviors, which makes them more applicable in encapsulating water insoluble bioactive compounds.^19^ Preparation of edible pickering emulsions are limited by a few food ingredients such as zein. Zein, the main protein in corn, is introduced as a good candidate for developing pickering emulsions.^20^ Zein is unstable in aqueous phases and it needs to be solubilized in an ethanolic solution (ethanol: water of 70:30 v/v%).^21^ Although the ethanolic solutions are regarded as safe, however, limited use of ethanol in industrial scale could provide a better condition. To eliminate the precipitation of hydrophobic zein nanoparticles (ZN) and use of ethanolic solution in preparation of ZN, the present study was focused on the production of ZN with non-flammable polyethylene glycol (PEG 400). Thus, the aims of the current research were replacing ethanol with nonflammable polyethylene glycol (PEG 400) in producing ZN (*i*), preparing zein-CMC complex for stabilizing water in oil (W/O) emulsions (*ii*), and nanoencapsulation of rutin (as a sample nutraceutical/drug) with prepared protein-polysaccharide complex (*iii*).

## Materials and Methods

### Materials

Purified zein (>99%), Span 80 (S 80), and Tween 80 (T 80) were purchased from Merck Chemicals (Darmstadt, Germany). Rutin (Quercetin-3-rutinoside trihydrate, > 95% purity), Oleic acid, CMC, and polyethylene glycol (PEG 400) were obtained from Sigma Aldrich (St. Louis, MO, USA). All the ingredients are regarded as a safe and are included in the food additives list of FDA, which are allowed for direct use in foods. In this regard, T 80, a non-ionic water-soluble surfactant, is suitable for O/W emulsion stabilization. It is reported that the non-ionic surfactants, especially Tween series, have less irritation and toxicity in comparison to the ionic ones. The FDA (21CFR172.840) approved T 80 as a direct food additive. Acceptable daily intake (ADI) for T 80 is 25 mg/kg body weight/day and its LD 50 varies between 4500 to 63 840 mg/kg.^1^ Other chemicals such as oleic acid, zein, and CMC are generally well known for their nontoxic and safe application in foods. Polyethylene glycols specially with lower molecular weights are widely used in a variety of food and pharmaceutical formulations. Generally, they are regarded as nonirritant and nontoxic constituents and included in the food additives list of FDA.^22^

### Zein-CMC nanocomplex preparation

Pure zein (2 g) was dissolved in different ratios of ethanol: PEG 400 (v/v%) solutions using different temperatures, T 80 concentrations, and pH values (Table 1). The prepared solutions were then centrifuged at 7363 g and the supernatant, which contain separated ZN. The ethanol was removed then using evaporator. In order to provide Zein-CMC complex, various CMC concentrations (10%, 20%, and 40%) were added to the optimum formulation obtained from Table 1, regarding the particle size and particle efficiency, using magnetic stirrer for 20 minutes. The final Zein-CMC nanocomplex (ZCN) was used for encapsulation of rutin.

**Table 1:** Composition of zein nanoparticle formulations*

**Ethanol:PEG 400 (v/v%)**	**Tween 80 (%)**	**Temperature (°C)**	**pH**	Formulation*
0:80	0.5	30	3	F1
			6	F2
			9	F3
		45	3	F4
			6	F5
			9	F6
		60	3	F7
			6	F8
			9	F9
	1.5	30	3	F10
			6	F11
			9	F12
		45	3	F13
			6	F14
			9	F15
		60	3	F16
			6	F17
			9	F18
40:40	0.5	30	3	F19
			6	F20
			9	F21
		45	3	F22
			6	F23
			9	F24
		60	3	F25
			6	F26
			9	F27
	1.5	30	3	F28
			6	F29
			9	F30
		45	3	F31
			6	F32
			9	F33
		60	3	F34
			6	F35
			9	F36
80:0	0.5	30	3	F37
			6	F38
			9	F39
		45	3	F40
			6	F41
			9	F42
		60	3	F43
			6	F44
			9	F45
	1.5	30	3	F46
			6	F47
			9	F48
		45	3	F49
			6	F50
			9	F51
		60	3	F52
			6	F53
			9	F54

*All formulations include 20 (v/v%) water and 100 mg zein nanoparticles (PEG: polyethylene glycol).

### Nanoparticle efficiency

Freshly produced ZCN solutions (Table 1) were centrifuged at 7363 g for 15 minutes to separate any larger particles. The resulting precipitated phase were then dried and weighed. The particle efficiency (PE%) was calculated as follow (Eq. 1):

Eq. (1) $$PE\%=\frac{Total\;weight\;of\;ZCN-Weight\;of\;dried\;ZCN}{Total\;weight\;of\;ZCN}\times 100$$

### W/O/W multiple emulsion producing procedure

The multiple water in oil in water (W/O/W) emulsions were produced in two steps using oleic acid, S 80, rutin, ZN, and ZCN. Frist, formulations of preliminary W/O nanoemulsions were prepared using oleic acid, S 80, and ethanolic solution of rutin. Rutin (20 mg) was gradually added (1 mL/min) into the mixture of oleic acid and S80 (Oleic acid to S 80 ratio was 10:1) using magnetic stirrer and was given enough time to achieve a transparent W/O solution. Secondly, the prepared emulsions were added to the various outer ZN and ZCN solutions using a homogenizer (9803 g) for 5 minutes at 25±2°C (Heidolph, Kelheim, Germany) and then W/O/W were homogenized for further 15 minutes.

### Particle size, size distribution, and zeta potential

Polydispersity index (PDI, narrowness of the particle size distributions), zeta potential (surface charge of the particles) of rutin loaded ZCNs (R-ZCN), and particle diameter (Z-average) of R-ZCNs were investigated using a dynamic light scattering instrument (Zetasizer Nano ZS, Malvern Instruments, UK). To avoid any multiple scattering effects, specimens were diluted 50-fold using deionized water before size measurements and then size measurements were performed at 25 ± 2°C. All measurements were carried out in three replicates after overnight storage.

### Encapsulation efficiency (EE%) and stability (ES%) of rutin loaded ZCNs (R-ZCN)

In order to plot the calibration curve of rutin, different standard solutions were prepared using absolute ethanol (1, 5, 10, 15, 20, and 25 ppm). Then, the absorbance was read at the wavelength of 258 nm using the spectrophotometer (Ultrospec 2000, Pharmacia Biotech, England). Loading capacity (LC%), encapsulation efficiency (EE%), and encapsulation stability (ES%) were studied by centrifugation method. R-ZCNs (1 mL) were placed in a Millipore tube (cut off of 10 kDa, Millipore, Bedford, MA, USA) and centrifuged (Universal 320, Hettich, Germany) at 78 g for 10 minutes. The free rutin amounts in the filtrate phase was measured spectrophotometrically (258 nm). Encapsulated and non-encapsulated amounts of rutin were determined in the retentate filtrate phase, respectively. Hydroethanolic solution (50:50 v/v%) was used for evaluating free rutin in the retentate phase as rutin becomes insoluble and precipitates. The summation of encapsulated and non-encapsulated rutin in all samples was 100%, indicating that there was no free rutin retained by the membrane. The percentages of ES and EE were calculated as following equations (Eq.2 and Eq.3):

Eq. (2) $$EE\%=\frac{W_{TR}-W_{FR}}{W_{TR}}$$

Eq. (3) $$ES\%=\frac{W_{TR}-W_{FR}}{W_{ER1}}\times 100$$

Where, W_TR_ is the total quantity of rutin in emulsions, W_FR_ is the amount of free rutin in the filtrate phase, and W_ER1_ is the quantity of encapsulated rutin at the first day of emulsions production.

### Encapsulation stability at different pH values

The stability of R-ZCNs in different pH values was determined by the importing electrode of the pH meter (Coming, Herts, England) into the specimens. To avoid any temperature variations, the temperature of pH meter was kept the same as the specimens’ temperatures. The pH values of the samples were read at these fixed conditions.

### Morphology studies

The morphology of the optimum R-ZCN formulation was investigated by scanning electron microscopy (MIRA3 FEG-SEM, TESCAN, Czech Republic). Dry sample was needed for SEM, which was prepared by drying one droplet (100 μL) of optimum R-ZCN on an aluminum foil (1 cm × 1 cm) at room temperature. These dried specimens were coated with gold under vacuum condition in an argon atmosphere (DST1, Nanostructured coating co., Tehran, Iran).

### Statistical analysis

All experiments were carried out in three replicates and the mean values were reported. The SPSS software (version 18.0) was used for running a completely randomized design (CRD) using one-way ANOVA followed by Duncan’s mean comparison test at the 5% significance level.

## Results and Discussion

### Formulation characterizations

**Figure 1:**
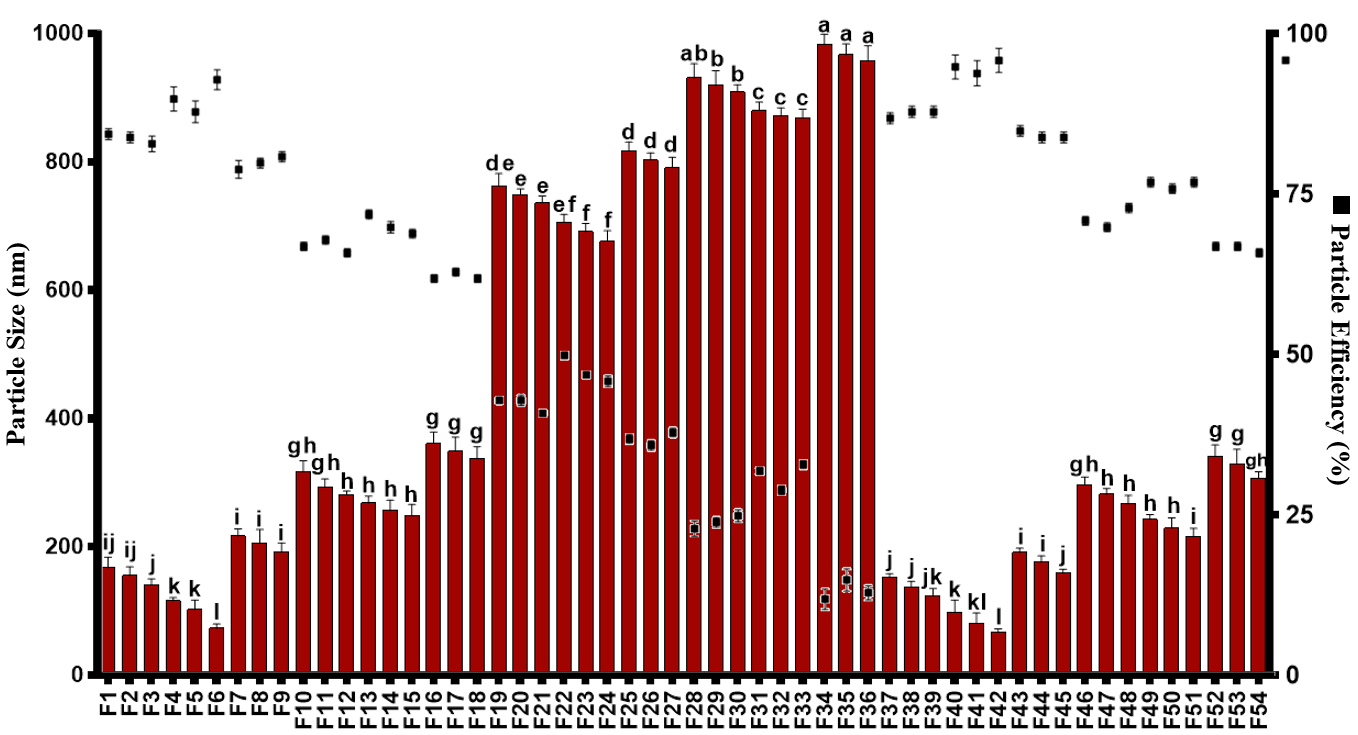
Particle size and particle efficiency (PE%) of initially developed zein nanoparticles.

Zein nanoparticles were produced using various conditions such as pH, temperature, and ethanol to PEG ratios, in order to achieve the optimum formulation and also asses the feasibility of ethanol substitution by PEG. The results of initially developed ZN (Table 1) were presented in Figure 1. According to Figure 1, solvent ratios played notable role and formulations containing ethanol: PEG ratio of 40:40 (v/v%) showed much larger particles than two other solvent ratios. Indeed, these results refer to this point that the preparation method of ZN plays an outstanding role in providing lower particle size. There are intensive reductions in particle sizes when two other solvent ratios (Ethanol: PEG ratios of 0:80 and 80:0 (v/v%)) used. This indicates that these two solvent ratios, which produced remarkably similar results, are good candidates for synthesizing ZN. The main point that can be figured out is the role of PEG. The results showed that ethanol can successfully be replaced by PEG, which can remarkably ignore any flammability during zein nanoparticle preparation processes. Temperature, pH, and concentrations of T 80 were other formulation parameters, which showed some effects on the particle size of initially prepared ZN. As it can be seen in Figure 1 there were other smaller jumps in graphs, which can be attributed to the effects of process temperature on the particle sizes. The results indicated that the formulations prepared at 45°C had smaller particle sizes than two other temperatures (30 and 60°C). This was founded in all formulation conditions (such as solvent ratios and etc.) indicating that the temperature can act as an independent parameter that is able to significantly affect the particle sizes. It was thought that, the denaturation behavior of the zein protein is responsible for these results. It seems that at lower temperatures (30°C) zein is maintained in its nature form that might cause lower solubility and consequently prevent the particles to be better dispersed.^23^ At 60℃, the more denaturation can make the larger and more liner form of zein, which might then cause particle aggregation during the process. At 45℃, the partial denaturation of zein particles might cause better solubility and lower aggregation during the preparation process.

The concentration of T80 was also affected the particles sizes as well as pH values. It can be understood from Figure 1 that, in general, the lower concentrations of T 80 (0.5%) showed better effects on size reduction than 1.5% ones. This can be attributed to the partial coalescence phenomenon, in which, at higher concentrations this phenomenon can be occurred that consequently cause larger particles. Although the application of T 80 reduced the effects of pH on the particle sizes (due to its nonionic nature), there are still some significant effects observed in the formulations. The formulations prepared at higher pH values (pH=9) showed better results than pH values of 3 and 6. It was reported that zein tend to make films at acidic conditions, which can be the main reason for these larger particles.^24^

### Zein nanoparticles efficiency

Particle efficiency (PE%) is defined as the amounts of non-precipitated nanoparticles derived from their solution. The PE% of the initially developed ZN (Table 1) was reported in Figure 1. As it can be seen, the ethanol: PEG ratio of 40:40 (v/v%) caused lower PE% than two other ratios. The maximum PE% was achieved when the solvent was PEG or ethanol. The results indicated that the formulations providing smaller particles had a high PE%. In fact, nano-dimensional size prevented the particles from aggregation phenomena. In this case, formulations F6 and F42 had significantly higher PE% values. F6 and F42 were produced using ethanol: PEG: water ratios of 0:80:20 and 80:0:20 (v/v%), respectively. The semi similar results of the formulations based on PEG with ethanol ones confirms the results in size analyzing part. It shows that PEG not only produced smaller particle sizes, but also is able to provide higher PE% values. These results might be attributed to the viscosity of PEG, in which the PEG not only acts as a good solubilizer, but also prevents the particles from aggregation. It is clearly observed that there was a special trend in PE% reduction in which, the formulations obtained in acidic conditions showed unstable features resulting in particle aggregation and precipitations that significantly reduced the PE% values. F6 and F42 showed PE% of 93±1.54 and 95±1.76, respectively. It indicates that PEG is able to maintain 1.86 g of zein in nano dimensional scale. Regarding the results obtained from size and PE% assessments, formulations F6 and F42 were chosen for further assessments.

### Zein-CMC nanoparticles

**Figure 2:**
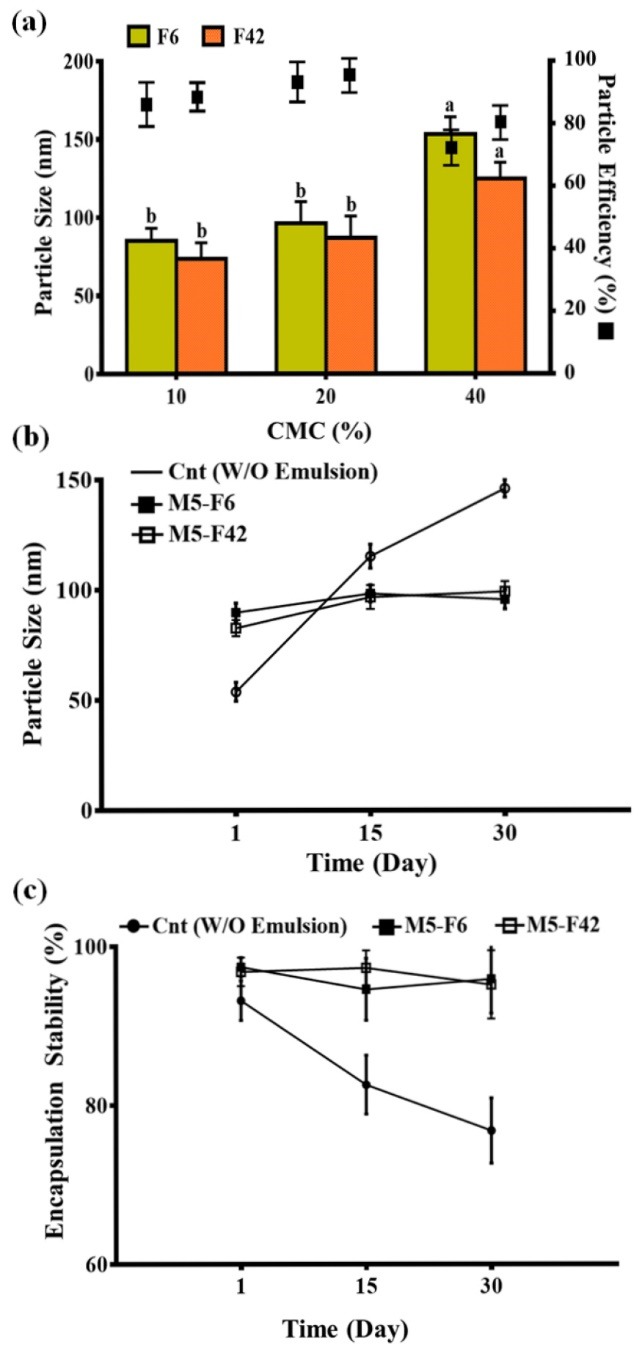
Particle size and particle efficiency (PE%) of zein- carboxymethyl cellulose (CMC) complexes (a), particle size (b) and encapsulation efficiency (EE %) (c) of water in oil (W/O) emulsion and water in oil in water (W/O/W) multiple (M) emulsions based on F6 (M5-F6) and F42 (M-F42).

Zein-CMC complexes were produced by incorporation of different concentrations of CMC to the ZN. The results are shown in Figure 2. Concentrations of CMC were assessed regarding the particle size and PE% values. According to the Figure 2a, the results for PEG and ethanol did not showed any significant differences, which furtherly proof that the PEG can be a deserve substitution for ethanol. However, the particle sizes were non-significantly increased when CMC was incorporated. The concentration of 20% CMC showed the smallest highest particle size (<100 nm). Although the 10% of CMC produced smaller particle sizes, it showed lower PE% values, which indicates that lower amounts of particles maintained their non-precipitated nano-dimensional sizes. The reason might be attributed to the lower amounts of CMC (10%), providing insufficient viscosity to prevent the particle aggregation. Furthermore, it might produce insufficient CMC-protein interactions resulting in lower barrier properties against particle aggregations. The higher CMC concentrations (40%) increased the particle sizes. The reason might be attributed to this point that CMC at higher concentrations make the intense interactions with zein proteins resulting in larger particles and higher precipitation. At this manner, CMC not only did not act as a barrier, but also it enhanced the aggregation phenomenon. The aggregation phenomenon might be attributed to the bridges generated between CMC and zein proteins. Thus, it was taught that CMC could present its barrier properties just in lower concentrations and can act as a particle aggregation promotor at higher concentrations. These results were in line with others, indicating that CMC has positive effects on size reduction.^25^ It was reported that using CMC reduced the zein particle sizes from 250 nm to 113 nm.^23^

### W/O/W multiple emulsions

In order to evaluate the W/O/W emulsions, the effects of ZN and ZCN solutions on the prepared O/W emulsions were studied (Table 2). Particle size of prepared formulations showed that the W/O/W emulsions doubled in size once ZN and ZCN added to the initial W/O emulsions. However, this increase was less in ZCN samples in comparison to the ZN ones. Among the multiple (M) W/O/W emulsions prepared with ZN, M1 showed higher size than two others (M2 and M3). For example, regarding the same amounts of ZN (M1-F6 and M1-F42) and ZCN (M4-F6 and M4-F42), ZCN based W/O/W emulsions showed smaller particle sizes, which might be attributed to the keeping factor properties of them. In other words, applied CMC played a positive role in fully covering the surfaces of particles. Furthermore, it also can provide better steric hindrance. Results in Table 2 showed that the W/O/W emulsions prepared with 50% ZN and ZCN (M2 and M5) provided better particle sizes. This might be related to the sufficient surface coverage of nanoparticles. However, the differences between the M2 and M5 ones, can be expressed by the capability of ZCN solution to better fulfill the surface and make strong barrier against the particle aggregation phenomena. These results also declare the nonsignificant differences between samples produced by PEG and ethanol. Finally, M5 (in both F6 and F42) was chosen to the further investigations due to its lower particle size and higher zeta potential values.

**Table 2:** Experimental design of prepared multiple water in oil in water (W/O/W) emulsions

	**ZN (mL)**	**ZCN (mL)**	**W/O Emulsion (mL)**	**Particle Size (nm)**	**Zeta Potential (-mV)**
Cnt	-	-	100	53.8±4.3^e^	26.9±1.1^g^
M1-F6	25	-	75	146.4±2.7^a^	31.4±0.3^f^
M2-F6	50	-	50	121.7±4.8^b^	33.7±0.1^e^
M3-F6	75	-	25	125.2±6.3^b^	36.7±1.2^d^
M4-F6	-	25	75	102.8±4.6^c^	46.8±1.3^c^
M5-F6	-	50	50	89.8±4.2^d^	49.2±0.4^b^
M6-F6	-	75	25	119.4±5.8^b^	52.6±0.8^a^
M1-F42	25	-	75	140.7±3.9^a^	31.4±0.3^f^
M2-F42	50	-	50	118.3±2.5^b^	33.5±0.4^e^
M3-F42	75	-	25	120.7±1.7^b^	36.4±0.8^d^
M4-F42	-	25	75	95.4±5.3^d^	47.3±1.3^c^
M5-F42	-	50	50	83.7±3.7^e^	48.4±0.6^b^
M6-F42	-	75	25	115.6±6.4^b^	51.6±0.7^a^

Abbreviations: ZN, Zein Nanoparticles; ZCN, Zein-CMC Nanoparticles; CMC, carboxymethyl cellulose; Cnt: control water in oil (W/O) emulsion without any ZN or ZCN; M1-F6: Multiple (M) W/O/W emulsion containing F6 ZN. The data were presented as Mean±SD and the different superscripts indicates significant differences at 5% Duncan’s test.

### Releasing profile

**Figure 3:**
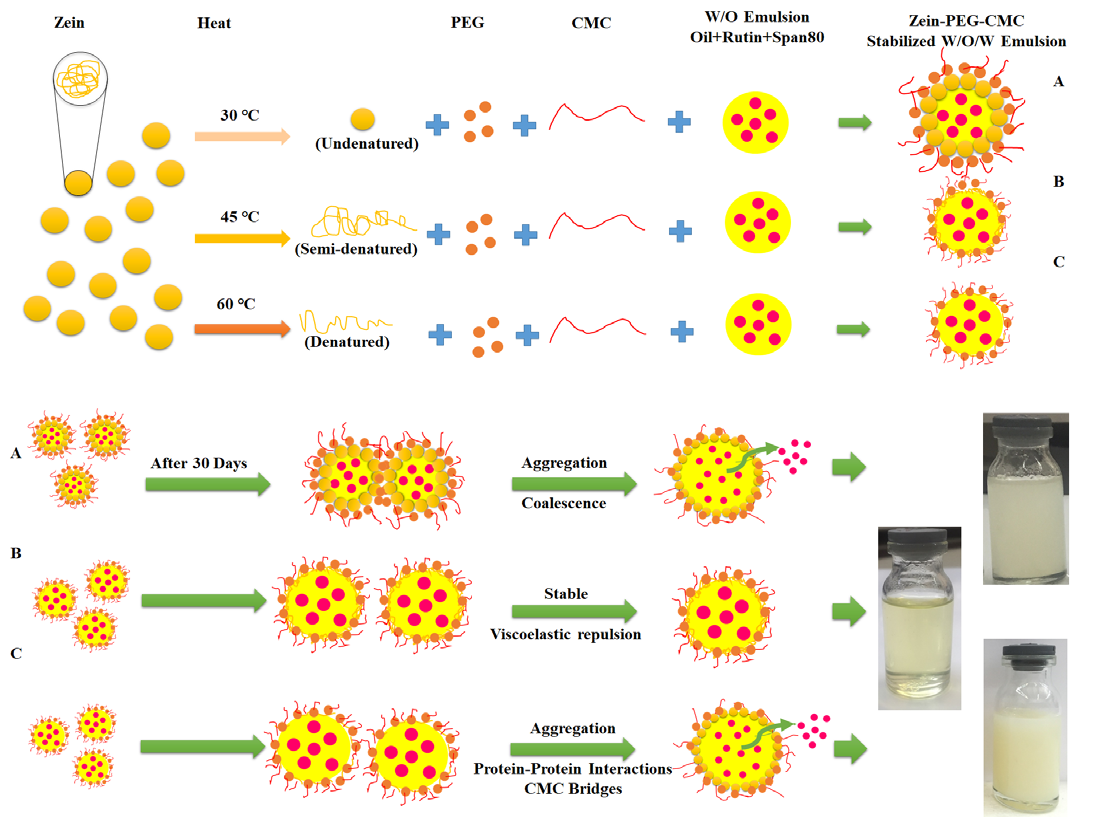
Schematic presentation of prepared water in oil in water (W/O/W) multiple (M) emulsions.

The W/O/W emulsions containing ZCN in their structure (M5-F6 and M5-F42) were used to be evaluated during the storage time (30 days) at refrigerator. The particle size and ES% of the emulsions were presented in Figure 2. As shown in Figure 2b, the W/O emulsion had increasing trend in particle size during 30 days of storage, while two others maintained their size stability. This indicates that the prepared W/O/W emulsions provided better physical stability, which can be expressed due to the use of CMC. It is taught that although the CMC increased the particle size, it enhanced the stability of the particles through viscoelastic repulsions. Another reason for higher physical stability of W/O/W emulsions can be related to their higher zeta potentials, which can provide enough electrostatic repulsive forces between the particles. Furthermore, the amounts of rutin leakage from the W/O nanoemulsions was remarkably more than the W/O/W ones, indicating the positive effects of applied conditions in their formulation (Figure 2c). The higher EE indicates lower release rate. In dead, the W/O/W emulsions kept the rutin in their core much better than individual W/O ones during 30 days of storage. In another word, the used PEG-CMC provided better structural integrity resulting in long lasting ES. These results are remarkably comparable with other researches. In a study for encapsulation of essential oils using ZN, it was shown that releasing rate was more than 70% during 50 hours.^26^ It was reported that using CMC can provide better releasing profiles.^25^ The important parameter in using CMC is its concentration. Using higher CMC concentrations, as founded in this study, could have adverse effects on the releasing profile. To better explain the stability, the schematic presentation of prepared W/O/W emulsions were illustrated in Figure 3.

### pH evaluations

The stability of the prepared emulsions at different pH conditions was evaluated using pH values of 1.2, 6.8, and 7.4 for stomach, intestinal, and colon, respectively. The acidic and neutral pH values were adjusted using HCl 0.1 N. and buffer phosphate, respectively. In this part, M5-F6 W/O/W emulsions, which showed better results, were evaluated and the results were shown in Table 3. The results showed that at acidic pH values (1.2) particle size of all samples increased significantly. However, the increase in the W/O/W emulsions sizes was in lower rate than individual W/O ones, indicating that W/O/W emulsions were more resistance against pH changes. These findings might be due to the behavior of the zein proteins in the presence of acid conditions. While, using the CMC provide some structural enhancements that make the zein based nanoemulsions more stable against pH changes. ES of the samples was affected by the pH changes, as well. The negative effect of acidic pH values might be expressed by the particle aggregation, which leads to release and more leakage of encapsulant due to lack of ability of nanoemulsions in retaining their encapsulant. These results also provide this fact that these types of W/O/W emulsions can be used for food fortifications especially those with higher pH values such as milk. It was reported that the ZN are stable at pH values higher than 8,^24^ while in this study we found them to be physically stable at pH values lower than 8. This can be explained by the modifying effects of the PEG and CMC on the pH based behavior of the ZN.

**Table 3:** Particle size and encapsulation stability (ES%) of rutin loaded water in oil in water (W/O/W) emulsions at different pH values

**Emulsion/Day**	**Particle Size (nm)**			**ES%**		
	**pH**			**pH**		
	**1.2**	**6.8**	**7.4**	**1.2**	**6.8**	**7.4**
Cnt/1*	65.6±7.36^f^	58.6±8.83^f^	60.6±6.16^f^	89.23±5.27^ab^	90.17±4.66^ab^	92.29±4.66^ab^
Cnt/15	185.6±8.63^b^	128.3±7.86^d^	108.3±7.06^e^	74.64±6.16^b^	81.24±5.34^b^	83.53±6.1^ab^
Cnt/30	298.3±10.32^a^	168.4±9.57^b^	143.63±7.13^c^	63.74±8.32^c^	69.52±7.2^c^	68.02±6.04^c^
M5-F6/1**	93.6±8.73^e^	91.7±5.68^e^	86.18±7.08^e^	96.24±6.18^a^	97.57±2.1^a^	95.86±3.9^a^
M5-F6/15	98.6±6.29^e^	97.9±8.42^e^	92.09±6.74^e^	90.25±6.83^ab^	94.6±3.03^a^	94.08±5.3^a^
M5-F6/30	118.6±9.3^d^	96.26±7.41^e^	97.6±9.38^e^	86.63±6.19^ab^	91.54±3.89^ab^	97.13±2.39^a^

*Cnt: Control water in oil (W/O) emulsion without any zein nanoparticles or zein-carboxymethyl cellulose nanoparticles (ZCN), **M5-F6: multiple (M) W/O/W emulsion containing with F6 ZCN. The data were presented as Mean±SD, and the different superscripts indicates significant differences at 5% Duncan’s test.

### Morphology evaluations

**Figure 4:**
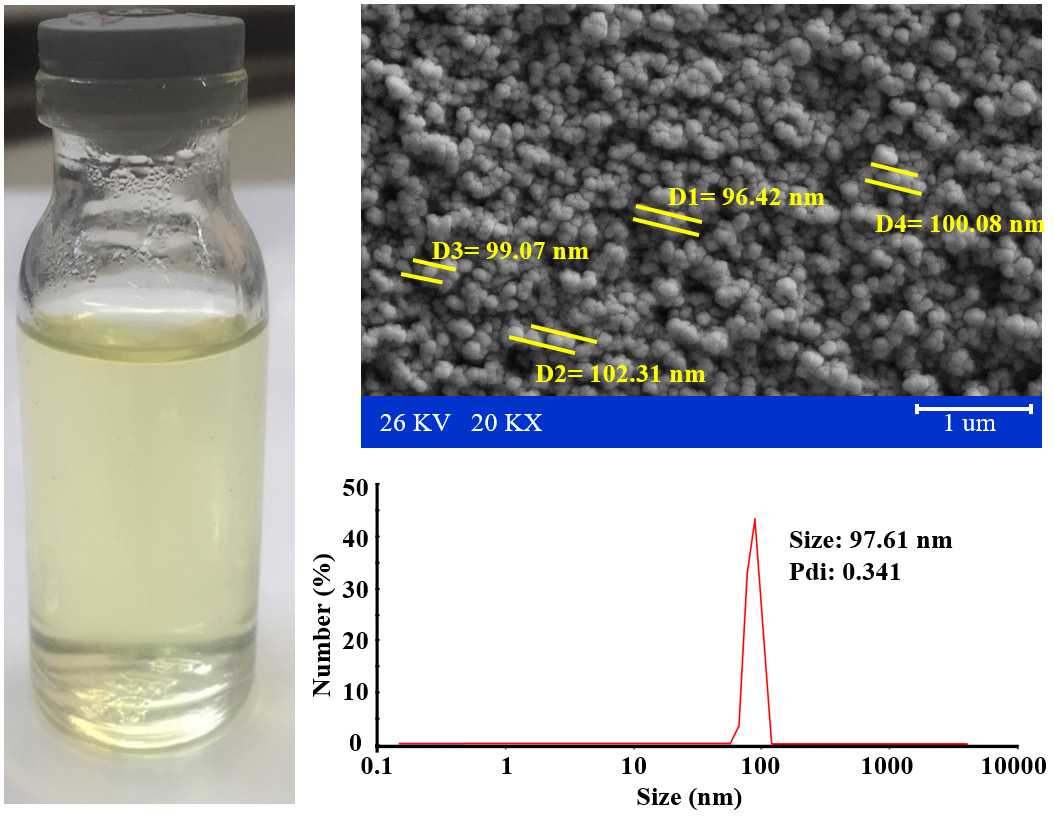
Scanning electron microscopy (SEM) and particle size analysis (Malvern) of water in oil in water (W/O/W) multiple (M) emulsions based on F6 (M5-F6) after 30 days of storage.

Scanning electron microscopy was applied for study the morphology of the samples. The morphology studies indicated that the W/O/W emulsions were almost round shape with smooth surfaces (Figure 4). The results obtained from scanning electron microscopy confirmed the DLS size analyzing results. Figure 4 indicates that after 30 days of storage, prepared W/O/W emulsions were still transparent due to their nano-dimensional particles. The shape and surface of the prepared W/O/W emulsions were in a good match with other researches, providing that these new formulation parameters didn’t affected the morphological characteristics.^27^

## Conclusion

The present research evaluated various parameters to develop food grade W/O/W delivery systems. Among the formulation parameters, solvent ratio intensively affected the particle size. Although incorporation of CMC slightly increased the particle sizes, it enhanced the particle efficiency as well. The results revealed that the presence of CMC in the formulation of W/O/W emulsions provided better particle sizes, enhanced encapsulation parameters, lower releasing rate, and longer period of physicochemical stabilities. As semi-denatured proteins have more bioavailability, semi-denatured zein protein not only make it suitable for making stable W/O/W emulsions, but also provide healthy and more bioavailable food grade emulsions.

## Ethical Issues

Not applicable.

## Conflict of Interest

The authors declare no conflict of interest.

## Acknowledgments

This research was supported by Drug Applied Research Center, Tabriz University of Medical Sciences, Tabriz, Iran, and Macquarie University, Sydney, Australia.
